# Early-Life Multilingualism And Midlife Cognitive Health: Evidence From High School And Beyond

**DOI:** 10.1093/geroni/igaf122.662

**Published:** 2025-12-31

**Authors:** Yue Qin, Eric Grodsky, Chandra Muller, John Warren

**Affiliations:** University of Wisconsin–Madison, Madison, Wisconsin, United States; University of Wisconsin–Madison, Madison, Wisconsin, United States; The University of Texas at Austin, Austin, Texas, United States; University of Minnesota, Minneapolis, Minnesota, United States

## Abstract

Learning a second language is cognitively stimulating, as it challenges the brain to make sense of an additional set of symbols and rules of syntax. However, little is known about how the timing and context of second language learning affect adulthood cognition. Drawing on the cognitive reserve theory and data from the 1980, 1982, and 2021 waves of High School and Beyond:1980 (N ≈ 13,980), we examine whether learning a second language before school or college, or taking a second language course in high school is associated with midlife cognition. We also examine potential differences in associations by race and ethnicity. We estimate linear regression models of composite cognition scores derived from multiple cognitive performances such as word recall, phonemic fluency, and semantic fluency, controlling for individual sociodemographic attributes. We find that being multilingual before college rather than before school was significantly linked to better midlife cognition before controlling for high-school academic performances, with larger benefits for Hispanics. This is possibly because learning English promotes the adaptation of Hispanics, whose first language is often Spanish, in the US educational system; Hispanics also have more opportunities to navigate different linguistic contexts between school and home. Taking second language course(s) in high school was also associated with better midlife cognition, with no significant racial or ethnic differences in the association. This study contributes to the growing research on the early-life factors influencing adulthood cognitive health inequality, highlighting the importance of the timing and context of second language learning in shaping cognitive outcomes.

